# Propolis Diterpenes as a Remarkable Bio-Source for Drug Discovery Development: A Review

**DOI:** 10.3390/ijms18061290

**Published:** 2017-06-17

**Authors:** Noushin Aminimoghadamfarouj, Alireza Nematollahi

**Affiliations:** Faculty of Pharmacy A15, The University of Sydney, Sydney NSW 2006, Australia; nami1357@uni.sydney.edu.au

**Keywords:** propolis, diterpenes, anticancer, antibacterial, Brazilian propolis, Mediterranean propolis, European propolis

## Abstract

Propolis is one of the complex, but valuable, bio-sources for discovering therapeutic compounds. Diterpenes are organic compounds composed of four isoprene units and are known for their biological and pharmacological characteristics, such as antibacterial, anticancer, and anti-inflammatory activities. Recently, advancements have been made in the development of antibacterial and anticancer leads from propolis-isolated diterpenes, and scrutiny of these compounds is being pursued. Thus, this review covers the progress in this arena, with a focus on the chemistry and biological activities of propolis diterpenes. It is anticipated that important information, in a comprehensive and concise manner, will be delivered here for better understanding of natural product drug discovery research.

## 1. Introduction

Honey bees have been known to humans for more than 15,000 years, and archaeological studies have revealed rock paintings describing bees and hive beekeeping, and how human beings domesticated wild bees and obtained benefits from them, using beekeeping apparatus [1,2]. There is evidence regarding the frequent usage of the term “bee king” by ancient Egyptian kings [3]. Ancient physicians and priests exploited from bees products to protect their gods and holy places, cure patients, and especially honey for themselves to remain in good health [4]; also, there are materials in holy books about the benefits and positive aspects of using honey and bees for the human body [5]. Moreover other honey bee hive products, such as propolis and royal-jelly, have been extensively used in traditional remedies all around the world since early human history [6].

Propolis (bee glue), the resinous material that can be seen in different colors, is mostly collected by honey bees (*Apis mellifera* L., belonging to the family of Apidae, have been studied extensively for their behavior, morphology, and physiology [7]) from bark cracks and leaf buds of various types of plants. Bees carry propolis to the bee hive where they use this dark adhesive substance to seal the walls of their hive to fortify the skeletons and structures of combs, and also to mummify successful intruders’ cadavers which bees have killed inside but cannot convey out of their hive to prevent their decomposition [8,9]. Propolis enables bees to protect their colony against hive invaders by minimizing the hive entrance size. Additionally, bees can preserve their society against several diseases, such as molds and bacterial infections, through the antimicrobial and antifungal properties of propolis [8]. In linguistics the term propolis originates from the Greek pro (for “in front of”, “at the entrance to”) and polis (“city” or “community”) [10].

Meyer has described how the bees’ leg movement is actively involved in the propolis collection procedure, along with assistance from the bees’ mouth parts, tongue, mandibles, and corbiculae. Once pollen baskets on the bee hind legs get full, bees will fly back to hive where propolis removal is carried out by mainly older bees, whose wax glands have been almost atrophied, while younger bees are busy building combs and capping cells for honey [11]. Three main theories have been discussed to highlight the factors affecting propolis collection by bees; firstly, the availability of propolis in the hive; secondly, the climate and seasonal changes; and, thirdly, some innate changes happening in the propolis foragers’ performance by late summer [12]. Some breeds of bees collect propolis more than others; for example, the grey mountain Caucasian honey bees have the highest activity in propolis collection [13], whereas some species and varieties of honey bees show very little interest in propolis and almost make no use of it, such as tropical honeybees (*Apis cerana*, *Apis florae*, and *Apis dorsata*) and African *Apis mellifera* [14].

Propolis melting point is known to be around 65 °C, but in some samples it goes higher, up to 100 °C [15]. About half of the propolis is composed of resinous materials, and other main constituents are as follows: wax, essential oils, and pollen [16]. The chemical groups of compounds identified in the propolis sample include flavonoids, aliphatic acids and esters, aromatic acids and esters, chalcones, terpenes, lignans, stilbenes, prenylated stilbenes, prenylated benzophenones, benzofuran, and sugars [16,17,18]. In recent years, there have been several studies done on single isolated compounds from propolis [18,19]. The chemical composition of the propolis varies based on its botanical origin. Propolis collected from different botanical regions exhibits different chemical outlines. There is no information showing that bees can engage any chemical process on the collected resins [20].

Propolis has a strong background use in human history, and around 400 years ago it was formally accepted as a medication by pharmacopoeias [21]; however, it was not until the last century that propolis popularity soared in European societies owing to its antibacterial characteristics. In modern times, propolis has been recommended by herbal specialists to manage and overcome infections, dermatitis, and gastroduodenal ulcers. In recent decades propolis is known as a popular complementary medicine in various dosage forms, such as lozenges, creams, and mouthwashes. Moreover, it enters into the cosmetic industries as a unique natural constituent [10].

Diterpene, a type of terpene, is one of the outstanding chemical structures inside propolis and has shown a broad array of biological effects, such as antibacterial, antioxidant, anti-inflammatory, antifungal, antiplatelet, anticancer, and antihypertensive activities [22,23,24,25,26,27,28,29,30,31,32,33]. Diterpene forms the primary skeleton chemical structure of many biologically-important natural compounds. Likewise, regarding the drug discovery rules, by assistance of medicinal chemistry, structure activity relationships, and semi-synthesis techniques, these isolated compounds have a sufficient potential to be used in drug development [34,35,36,37,38].

Herein, before focusing on the properties of diterpenes isolated from propolis, a concise summary of propolis pharmacological and biological activities is presented and then, in this review, we discuss the biological and pharmacological activity of the diterpenoid propolis along with their chemical structures, sources, and their probable action mechanisms, to display the potency of such naturally-occurring organic molecules as novel resources for future drug discovery.

## 2. Propolis Biological Activity

### 2.1. Antibacterial, Antiviral, Antifungal, and Antiparasite Activities

The very first data published regarding the antibacterial activity of propolis extract dates back to 1980, showing that sensitivity of *Streptococcus* species to propolis extract was reported [39]. Later, the alcoholic extract of propolis effectiveness was remarked against a *Bacillus* strain [40] and growth inhibition activity (at 3 mg/mL) was recorded against *Pseudomonas aeruginosa* and *Escherichia coli*, even though no activity was observed for propolis extract on *Klebsiella pneumoniae* [16,41,42]. Antibacterial synergistic effect was seen by alcoholic extract against *Staphylococcus aureus* and *Escherichia coli* when tested simultaneously in the medium with other antibiotics [43,44].

Formation of Flu viruses (A and B types) are affected by propolis [45]. Herpes virus counts were dramatically reduced by using propolis (30 μg/mL); however, less inhibition was seen against adenoviruses [46]. The observed antiviral activity for propolis originates from its complicated chemical compositions [47,48,49,50,51], and it was also reported that propolis can affect the cell receptors at the viral adsorption step [52].

Propolis antifungal activity was tested on *Trichophyton* and *Mycrosporum* species, along with propylene glycol solution, and exhibited synergistically-increased antifungal activity [53]. The same result was obtained using propolis along with other antifungal drugs on *Candida albicans* [54,55,56]. According to Fernandes Junior et al., propolis extract was examined on several fungi strains *(Candida species*) and based on the results more than 95% of tested strains were sensitive to propolis ethanol extract in concentrations less than 5% [16]. Propolis is reported to inhibit the growth of *Trichophyton verrucosum* at concentrations of 5% and 10% [57]. Antifungal activity of propolis ethanolic extract and its four different fractions against *Penicillium italicum* were assessed and obtained results showed all tested samples having strong antifungal properties, especially the ethyl acetate fraction [58].

Ghanaian propolis was evaluated for antiparasitic effects on *Trypanosoma brucei,* which causes the human sleeping sickness predominately in sub-Saharan Africa [59]. Propolis has showed complete inhibitory effects on the *Fasciola gigantica* eggs (at 200 μg/mL) [60]. According to Higashi et al., propolis strongly inhibits proliferation of *Trypanosoma cruzi* at 15 μg/mL [61]. In 1988, propolis was promisingly proposed to manage giardiasis (causing diarrhoea) as a natural source with the benefit of showing a minimum level of side effects during treatment [62]. The alcoholic extract propolis can terminate the proliferation of protozoa, such as *Toxoplasma gondii* and *Trichomonas vagilanis*. The extract at the concentration of 150 mg/mL showed lethal effects on three strains of *Trichomonas vaginalis* [63]. Propolis showed coccidiostat activity on *Chilomonas paramecium* [16]. Propolis ethanolic extract could strongly inhibit the growth of *Giardia lamblia* during the in vitro assay at the concentration of 11.6 µg/mL [64]. Antifungal and anthelmintic activities have been reported from Argentinian propolis ethanolic extract samples [65]. Likewise, for the antifungal activity from diterpenoid propolis, there is a mixture which decreased the adhesion of fungi to surface and has been used as a dental medicine [66].

### 2.2. Anti-Inflammatory Effect

Chinese and Brazilian propolis samples were studied for their anti-inflammatory mechanism of action. Both samples could affect and alter lipoglycan- and endotoxin-based inflammatory cascade in rodent macrophages. The in vitro experiment results confirmed that propolis extract decreases nuclear factor-κB (NF-κB) stimulation and suppresses the synthesis process of ubiquitin units. To conclude, although the alcoholic extract from China has dramatic differences compared to the Brazilian extract, both samples showed anti-inflammatory properties by blocking NF-κB function [67]. Argentinian propolis ethanolic extracts showed in vitro anti-inflammatory activity by reducing lipoxygenase and cyclooxygenase activities and nitric oxide production (by decreasing inducible nitric oxide synthase protein expression) [65]. Brazilian red propolis was analysed and anti-inflammatory and antinociceptive activities were observed through in vivo models [68]. Propolis samples collected from Chile were analysed for their phenolic profile and anti-inflammatory activity. The samples exhibited anti-inflammatory activity through inhibitory effects on nitric oxide release [69]. Nepalese propolis suppressed the interleukin-33-induced messenger RNA expression genes and established its anti-inflammatory effects in such a mechanism of action [70].

### 2.3. Cytotoxic Effect

According to Haldon et al., in 1980, fractions of propolis exhibited cytotoxic properties (at 2.6–3.3 μg/mL) on HeLa (human cervical carcinoma) and on human KB (nasopharynx carcinoma) cell lines. This result was confirmed by Ban et al., in 1983 [16]. The red propolis from Brazil has been reported by Awale et al. to possess cytotoxic activities [71]. Greek propolis showed anti-proliferative activity against human colon adenocarcinoma cells (HT-29) [72]. Brazilian propolis samples have cytotoxic activities against human hepatocellular carcinoma cell lines [73], and also the in vivo assay cytotoxic effects were recorded on mouse skin tumours [74].

### 2.4. Immunomodulatory Action

There is a study about propolis constituents which suppress T-lymphocyte cells but, conversely, can make macrophage function active. The same effect has been claimed for Brazilian propolis [75].

Propolis can affect intrinsic immunity through activating the immune response by increasing the production of cytokines, and elevating the level of expression of Toll-like receptors in spleen cells and macrophages [76]. Some propolis constituents can stimulate chemotactic activity in neutrophil cells. These propolis substances improve neutrophil migration function, which increases the ability of intra-cellular phagocytosis of white blood cells. A partially-purified propolis extract from Argentina showed significant chemotaxis elevation effects on the human immune system [77].

### 2.5. Toxicity

The lethal dose (LD_50_) of propolis has been reported to be around 2000 mg/kg [78]. Later, it was reported that the LD_50_ was about 700 mg/kg for alcoholic propolis extract, while it is reported as 350 mg/kg for the ether solution of propolis by Russian researchers [12]. The carcinogenesis of propolis in rats by adding propolis at the dose of 1 mg/mL in rat’s drinking water was studied and no differences were observed in controls and treated animals [79]. Propolis dermatitis was first reported from apiarists (assumed as an occupational eczema), later as the usage of propolis developed other non-occupational incidences have also been added to the propolis usage cautions [80]. It was showed that different propolis types can produce different degrees of contact allergy. Propolis allergy has been considered to have high levels of sensitization among children [81,82]. In a comprehensive experiment on propolis, patch warnings were recommended in use of the propolis for dermatological purposes for young children [83].

## 3. Diterpenes from the Propolis

**Figure 1:**
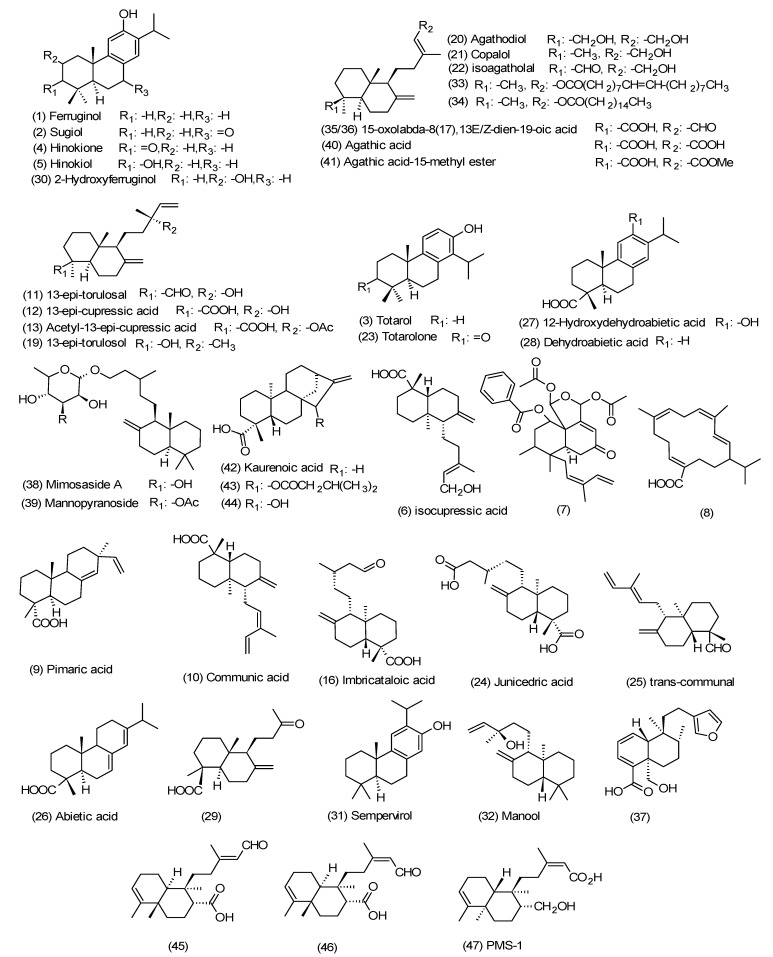
Structures of the isolated bioactive diterpenes from propolis as a natural source.

Diterpenes belong to the class of terpenes based on having the C_20_ skeleton, composed of four isoprene units originated from mevalonate or deoxy-xylulose phosphate (non-mevalonate) [84]. More than 3000 diterpenes have been explored from nature but only a small number of them have been recognized as clinically effective [85,86]. One of the rich resources of pharmacologically-active diterpenes in nature is propolis and these compounds sequestered from propolis might be used directly in treatment per their less toxic effects [87,88,89,90,91,92]. In this section, the propolis samples are composed of a high amount of diterpenoids (Figure 1), and their chemistry, biological, and pharmacological properties are discussed.

Tri- and di-terpenoids have recently been reported as the major constituents from an analytical study done on a propolis type collected from the southern part of Saudi Arabia. The majority chemical compositions of diterpenes of the propolis ethyl acetate (EA) fraction were compounds **1**–**5**. MTT cell viability assay exhibits that EA fraction have cytotoxic activity against Jurkat T-cells, A549 lung carcinoma, HepG2 liver cancerous, and SW756 cervix carcinoma cell lines with IC_50s_ in the range 1.8–6.3 μg/mL. Exploiting fluorescence microscope techniques, tubulins are recognized as the target for apoptotic properties of the propolis EA fraction, with high percentage content of terpenoids [93].

In a study on nanoparticle drug delivery system of Moroccan propolis, which endorses isocupressic acid (**6**), the diterpenoid, in high concentrations, having an antibacterial effect against methicillin-resistant *Staphylococcus aureus* (MRSA), was evaluated [94]. The result was consistent with the collected propolis from the northern part of Morocco (Bhalil) which followed the same proportional constituent pattern (diterpenoids have the highest share of its composition). The Bhalil sample exhibited inhibitory activities against amylase isozymes along with having substantial antioxidant activities [95].

A rare clerodanoid diterpene (**7**), accompanied by other established diterpenoids were characterized from Brazilian brown propolis, and it showed significant anticancer activities against a number of cell lines [96]. Compound **7** was initially reported from the same team as a patent possessing promising cytotoxic effects against LNCap cells to overcome prostate cancer with an IC_50_ of 6.2 μM [97].

Propolis samples from the central part of Chile were profiled and the extracts were biologically evaluated against Gram-negative strains, and antibacterial activities were observed. The existence of diphenylheptanoids and a diterpene (**8**) in collected samples might be responsible for such activities [98].

Characterization of Mediterranean propolis samples’ constituents from four different regions (Algeria, Greece, Croatia, and Cyprus), determined that Greek propolis composition, with antibacterial and antioxidant properties, is different from other common European propolis samples and has a higher percentage of diterpenoids (**3**, **6**, **9**, and **10**) while having lower amounts of phenolic compounds [99].

From studies on Libyan propolis, two bioactive diterpenes (**11**–**13**) were isolated, elucidated, and later their antiparasitic activities were evaluated to overcome African Trypanosomiasis. These diterpenoids showed almost the same activity with IC_50s_ of around 1.5 μg/mL against *Trypanosoma brucei*. Furthermore, these bioactive Libyan propolis constituents were studied for leishmaniosis, and they exhibited inhibitory activity against infection of macrophages with *Leishmania donovani* (IC_50s_ 5–22 μg/mL) [100].

In a chemical profiling of Saudi Arabian propolis samples, diterpenoids (**14**) and (**15**) were characterized and their botanical sources were identified as *Psiadia arabica* Jaub. et Spac and *Psiadia punctulata* DC., respectively. Compounds **14** and **15** were evaluated against local skin mycobacterium (*Mycobacterium marinum*) and sleeping sickness protozoan (*Trypanosoma brucei*) and both presented activities [101].

Propolis samples collected from different areas of Iraq were analysed and the results revealed there exist clerodanoids, a type of terpenes structurally similar to labdane diterpenes, in their constitutions. The samples showed antioxidant properties [102].

Exploiting gas chromatographic mass-spectroscopy (GC-MS), propolis samples having antibacterial activities collected from Malta were analysed, and numbers of diterpenes were categorized [91], previously published in another Mediterranean propolis type from Greece [90,91]. *Ferula communis* L. was proposed as this type of Malta propolis botanical source. The results showed the highest portions belong to compounds **3**, **6**, **9**–**11**, and **16**, while compounds **17**–**19** were in the minority [91]. Other diterpenoids found in this type of propolis are compounds **1**, **12**, **13**, and **20**–**31**.

Six diterpenes were isolated from Greek propolis in 2010. They demonstrated anticancer effects and, in an investigation done on this type of propolis, the isolated diterpenes had the activities against human colon adenocarcinoma cells (HT-29) with the lowest side effects on normal cells and introducing manool (**32**) (IC_50_ = 6.5 μg/mL) as the most active among them [72].

In the comprehensive study done on the Greek propolis from Cretan the diterpenes have been isolated and elucidated. Diterpenoids (**9**, **23**–**25**, and **33**–**36**) were reported for the first time from propolis. These compounds were tested against some Gram-positive and -negative bacteria. The results exhibited antibacterial activity. All tested compounds showed a broad spectrum of antibacterial activity, while diterpenes (**3**) and (**23**) had the highest activity against all examined bacteria. The minimum inhibitory concentrations (MICs) range of these compounds against the tested bacteria was from 0.07 to 1.80 mg/mL. Furthermore, the synergistic effect was noted for compounds **35** and **36** (*E* and *Z* configurations); hence, the combination of them had a profound activity against the Gram-positive bacteria [103].

In the study on the main botanical source of green propolis collected from Brazil, *Baccharis dracunculifolia*, for the first time a clerodanoid (**37**) was identified. Although this type of propolis showed antibacterial activity, compound **37** did not exhibit any significant antimicrobial properties [104]. The results were consistent with previous comprehensive liquid chromatography mass-spectroscopy (LC-MS) study on this type of propolis sample, plus the stated botanical source, shedding light on their chemical constituents profile, including the existence of diterpenoids (especially labdanoids) [105]. Extraction on Brazilian propolis samples showed diterpenoids were mostly found in methanolic extract, and only negligible amount of diterpenoids were traced in water extract; this is quite expected due to their non-polar characteristics [106].

Study on the European propolis extraction from Greece resulted in identification of diterpenoids **3**, **6**, **11**, **12**, and **19**–**22**. The isolated compounds were screened for their antibacterial and antifungal activities. They showed activity against both Gram-positive and -negative bacteria; however, compound **3** manifested strong antibacterial activity against Gram-positive bacteria, especially *Staphylococcus* spp, in comparison with references, which confirmed totarol’s (**3**) reputation as an antibacterial agent. Weak effects were reported from antifungal activity testings [107]. It is worth mentioning that a study of European propolis was directed to the isolation of diterpenes **6**, **10**, and **21** from an Italian propolis type with antimicrobial properties [108]. The same presence of diterpenoids in Italian propolis samples was also stated in a 2002 GC-MS analytical study on a Sicilian type [109].

The study on a Central America propolis type reported two glycoside diterpenes (**38** and **39**) sourcing from El Salvador propolis samples presenting the noteworthy antibacterial effects on *Staphylococcus aureus* with the minimum inhibitory dose almost three-times less than the obtained lethal dose from a toxicity bioassay [92].

Brazilian propolis anti-hepatotoxic methanolic extract resulted in the isolation of labdanoids (**12**, **13**, **40**, and **41**). In more details, compounds **12** and **40** showed the highest antihepatotoxic activities (IC_50s_ 80 and 45 µM, respectively), and this activity might be linked to diterpenoids’ healing effects observed on d-galactosamine/TNF-α-induced hepatic damage models [110,111,112]. The isolated compounds were also evaluated for the anti-helicobacter pylori activity, and compound **41** was active against all tested strains, while compound **13** limited its activity to only one strain [110]. There is a study which showed the isolated diterpenoid from Brazilian Meliponinae with antibacterial activity against Gram-positive bacteria, particularly against *Staphylococcus aureus*. Moreover, the samples from this type of propolis, which contained high concentrations of diterpenes, showed cytotoxic activities [113].

Interestingly, although most of reports about propolis samples’ bioactive constituents show *Apis mellifera* species play the key role in the sample collection procedure, a study reported tetra-cyclic diterpenoids (**42**–**44**) from propolis samples supplied by Brazilian native stingless bees (*Meliponini*). Compound **42** only showed antimicrobial property [114]. Two active clerodane diterpenes (**45** in *E* configuration and **46** in *Z* configuration) from Brazilian propolis had been isolated, elucidated, and assessed for their human hepatocellular carcinoma cell cytotoxicity [115].

Additionally, a promising antitumor diterpene (**47**) isolated and elucidated from Brazilian propolis showed significant effects, such as in vivo antitumor activity on mice skin [74], in vitro cytotoxic activities against hepatocellular, renal cell carcinoma, lung cancerous cells [116], and tumoricidal activity against HeLa 53 Cells (IC_50_ 87 μg/mL) [117].

As the first review report about diterpenoids sequestered from propolis, it is worth mentioning that labdanoids (**6**, **10**, **13**, and **16**) were purified from a Brazilian propolis type, with the same pattern for diterpenes found in *Araucaria* genus members, which provides a clue to its botanical source [118].

## 4. Discussions and Conclusions

Most diterpenes isolated from propolis possess antibacterial and cytotoxic activities; for that reason, in this section these two activities have been centred on and the mechanisms of action are discussed. Regarding the cytotoxic and anticancer characteristics of diterpenes obtained from propolis, some hypotheses have been generated. In the study about a diterpenoid from Brazilian propolis which had cytotoxic effects against human hepatocellular carcinoma, the growth of the malignant cells has been blocked by α-DNA polymerase inhibition [119]. Moreover, compound **47** (PMS-1) showed its antitumor activity through the inhibition of DNA synthesis. There are two pathways for this bioactivity. The first one is that by inhibiting the DNA synthesis in the de novo pathway, the occurrence of tumours has been decreased. In the second pathway, the salvage pathway, through reducing DNA synthesis, the growth of the tumours has been suppressed [74]. Furthermore, in the experiment on the cytotoxicity activity of manool (**32**), one of the most active diterpenes from Greek propolis, it had been exhibited that the cell cycle of the cancer cells was blocked at the G_2_/M stage [72]. The same mechanism has been reported for the propolis collected in Southern Brazil [120].

One of the known isolated diterpenes from propolis which had a significant antibacterial activity is totarol (**3**). Even though the mechanism of action of this compound is not clear but there are some proposed mechanisms for this activity [121]. One of these suggested mechanisms is that, the consumption of oxygen in bacteria cells is inhibited by this diterpene, and also totarol (**3**), can disturb the electron transport and respiratory pathway in the oxidation of bacteria membranes by inhibiting NADH-related enzymes, such as NADH-cytochrome C reductase, NADH-DPIP reductase, and NADH-CoQ reductase [122], although this hypothesis is not very robust regarding the activity of totarol (**3**) against anaerobic bacteria [123]. Moreover, there have been studies conducted on antibacterial activity against methicillin-resistant *Staphylococcus aureus* [124,125,126] and the main suggested mechanism for this activity is interfering with penicillin binding protein 2 expression [127]. This diterpene and its derivatives may affect the synthesizing of the adenosine triphosphate in bacteria [128], and also destabilizing the membrane integrity by decreasing the intermolecular forces of the bacteria phospholipid bilayer structure [129,130,131]. In 2007 it was stated that through inhibition of filamenting temperature-sensitive mutant Z (FtsZ) protein, the protein which moves to the division site throughout cell division in prokaryotic cells and is vital to construct a cell wall [132], the growth of the Gram-positive bacteria was blocked [133].

In summary, this review focused on biologically- and pharmacologically-active diterpenes obtained from propolis as the natural source. We have outlined the geographical locations of the recognized sources, and their bioactivities, plus the probable mechanisms of actions. Diterpene nuclei isolated from propolis are attractive for medicinal chemists to design and discover novel therapeutic agents owing to their less toxic side effects. By means of synthesis and applying the required changes in the diterpene core structures their bioactivities might be enhanced. For instance, by studying and synthesis of the different derivatives of totarol, alterations of the aromatic ring moieties, it was manifested that a hydroxyl moiety is crucial for existing antibacterial activity [134]. Furthermore, in vitro examinations illustrated that inserting moieties on the aromatic ring, apart from the hydroxyl group, decreases the antibacterial properties of this diterpenoid class [135]. Since the isolation of diterpenes from propolis gives a higher yield and easier access than the plant source, the isolated diterpenes can be used in the semi-synthesis of novel leads. As an example, the studies done on labdane-type diterpenes and clerodane diterpenes illustrated that the derivatives of these type of diterpenes can act as novel antimalarial, antileishmanial, and anti-inflammatory drugs [136,137]. Therefore, diterpenes are recognized and well-known to have a broad range of structures with different moieties which have significant effects on the critical medicinal targets for prevention and treatment of several diseases.

Despite the clear progress in natural products there are not enough in vivo studies on the claimed isolated diterpenes; thus, further in vivo examinations of these potent and safe agents are inevitable. Additionally, a systematic investigation of these type of compounds can be useful. To come to the point, this review is presented to display the importance of propolis as a novel and less toxic bioactive source of diterpenes.
